# Single-center experience of Ex vivo liver resection and autotransplantation for complex hepatic alveolar echinoccosis

**DOI:** 10.3389/fsurg.2023.1089788

**Published:** 2023-02-15

**Authors:** Jiaqi Yuan, Xiaobin Chen, Lizhao Hou, Haijiu Wang, Ying Zhou, Mingquan Pang, CaiRang YangDan, Zhixin Wang, Haining Fan

**Affiliations:** ^1^ Department of Hepatopancreatobiliary Surgery, Affiliated Hospital of Qinghai University, Qinghai, China; ^2^ Qinghai Province Research Key Laboratory for Echinococcosis, Qinghai, China; ^3^ Department of Gastroenterology, Xining Second People's Hospital, Xining, Qinghai, China

**Keywords:** ex vivo liver resection and autotransplantation echinococcosis, prognosis, complications, echinococcoses, China

## Abstract

**Objective:**

To summarize the single-centre experience of Ex vivo Liver Resection and Autotransplantation (ELRA) to treat end-stage hepatic alveolar echinococcosis (HAE).

**Methods:**

Retrospective analysis of clinical data and follow-up data of 13 patients admitted to the Affiliated Hospital of Qinghai University from January 2015 to December 1, 2020, with the Ex vivo Liver Resection and Autotransplantation for hepatic alveolar echinococcosis.

**Result:**

13 patients underwent successful total/ semi-ex-vivo liver resection combined with Ex vivo Liver Resection and Autotransplantation with no intra-operative deaths. the median standard liver volume was 1,118 ml (1,085–1,206.5 ml); the median residual liver volume was 634 ml (526.5–1,338 ml); The median weight of the autograft was 845.8 g (619.5–1,020.5 g), the median operation time was 14.5 h (11.5–16.15 h); the median anhepatic period time was 290 min (257–312.5 min). The median intraoperative blood loss was 1,900 ml (1,300–3,500 ml); the median number of erythrocyte suspensions entered was 7.5 u (6–9u). The median length of hospital stay was 32 days (24–40 days). Postoperative complications occurred in 9 patients during hospitalization,with 7 patients graded at grade III or higher by Clavien-Dindo; 4 patients died postoperatively. 1 patient had recurrent abdominal distension with massive thoracoabdominal fluid and coagulation dysfunction 8 months after surgery and was considered to have small liver syndrome. 1 patient developed HAE recurrence during the follow-up, which was considered intraoperative incisional implantation.

**Conclusion:**

ELRA is one of the most valuable therapeutic measures for the treatment of end-stage complicated hepatic alveolar echinococcosis. Precise preoperative assessment of liver function, individualized intraoperative duct reconstruction, and precise management of the postoperative disease can achieve better treatment results.

## Introduction

Hepatic Alveolar echinococcosis (HAE) is a zoonotic disease caused by echinococcosis infection. Since the disease has no specific symptoms in the early stages, most of them are already in the middle and late stages when they go to the hospital, and the lesions usually involve the first and second hepatic hilum and even erode the posterior inferior vena cava of the liver (1, 2); The multiple intrahepatic lesions with more severe destruction of intrahepatic structures have led to a relatively small number of patients, only 35%, undergoing radical hepatectomy (3, 4), Liver transplantation was recognized as the best treatment for patients with advanced hepatic alveolar echinococcosis because of the destruction of the liver and surrounding organs and the reduced prognosis for patients with such advanced disease (5). However, allogeneic liver transplantation can be associated with a shortage of donor's livers during treatment, and lifelong immunosuppressive medication, which leads to a significantly higher risk of recurrence of encapsulated worms and affects the use of liver transplantation in HAE (5, 6). Pichlmayr's team (7) first completed the resection of hepatic malignancies in 1988 using ELRA as a radical approach for tumours that conventional surgery could not remove. To overcome the dilemma faced by liver transplantation in HAE, in 2011, Professor Wen Hao's team (8) first reported the application of the isolated liver resection technique combined with ELRA to the treatment of HAE patients, which several centres subsequently adopted in China for the treatment of HAE with good results. The clinical data and follow-up data of 13 ELRA patients admitted to the Affiliated Hospital of Qinghai University from January 2015 to December 1, 2020, are retrospectively analyzed and reported.

### Patients and methods

Data of patients undergoing surgery: The data of 13 patients admitted to the Affiliated Hospital of Qinghai University from January 2015 to January 2020 who underwent ELRA for advanced hepatic vesicular encrustation disease were retrospectively analyzed, and their main characteristics were as follows: (1) the target lesion was challenging to resect *in vivo*, the reserved hepatic involved vasculature was difficult to resect and reconstruct, and bleeding from the liver and blood vessels close to the liver was difficult to control; (2) the prepared left hepatic vein-inferior vena cava venous confluence site invasion, pre-existing hepatic portal vein tertiary and higher branch invasion; (3) patient in the good physical condition and normal liver and renal function before surgery. There were 3 cases (23.1%) of males and 10 (76.9%) of females, aged 18–59 years, with a mean age of 38.3 years. Patients presented with symptoms of jaundice before surgery in 8 cases; recurrent upper abdominal discomfort in 5 cases; Indocyanine green retention rate at 15 min <10% in 6 cases and >10% in 7 cases; Child-Pugh A in 8 cases and grade B in 5 cases; all patients underwent preoperative PHI staging (HX-PHI Staging system) (9), The severity of vascular erosion was classified into 3 grades and different types according to the preoperative imaging assessment of the operated patients; the specific typing and grading levels are shown in Table 1. this study complied with the Declaration of Helsinki. All patients had postoperative pathology confirmed as hepatic echinococcosis; the patients and their families signed an informed consent form. The patient's preoperative clinical data and preoperative assessment results are shown in Table 2.

**Table 1:** Intraoperative information and postoperative outcomes of 13 ELRA patients.

Category	Numeric	Category	Numeric
**Operating time (h)**	14.5(11.5–16.15)	**Posterior peritoneal collateral circulation** (n,%)	
anhepatic period time(**min**)	290 (257–312.5)	Yes	5 (38.4)
**Residual liver volume (ml3**)	634 (526.5–1,338)	No	8 (61.6)
**Standard liver volume (ml3**)	1,118 (1085-1206.5)	**HX-PHI typing**	
**Bleeding volume (ml**)	1,900 (300-4,000)	I	4 (30.8)
**Infusion of red blood cells** (**U)**	7.5(3–20)	II	3(23.0)
**Reconstruction of the inferior vena cava of TPS in the hepatoless phase** (***n*,%)**		III	6(46.1)
Artificial/autologous revascularization	7 (53.8)	IV	0 (0)
Artificial vessel temporary replacement	4 (30.8)	**Influx tract invasion typing** (***n*,%)**	
PV-subhepatic IVC dissection end anastomosis TPS	2(15.4)	1	2(15.4)
**IVC reconstruction material** (***n*,%)**		2	7(53.8)
Autologous vessel	6 (46.1)	3	4 (30.8)
Allogeneic vessels	2 (15.4)	**Hepatic vein invasion subtype** (***n*,%))**	
Artificial vessel	4(30.8)	1	4(30.8)
None	1 (7.6)	2	9 (69.2)
**PV reconstruction** (***n*,%)**		**Inferior vena cava invasion staging** (***n*,%)**	
LPV-PV end-to-end anastomosis	10 (76.9)	1	1 (7.6)
PV end-to-end anastomosis	2 (15.4)	2	12 (92.4)
None	1 (7.6)	**Type of complications** (***n*,%)**	
**HA reconstruction** (***n*,%)**		None	4 (30.8)
LHA-PHA end-to-end anastomosis	6 (46.1)	Bile leakage	2 (15.4)
PHA-PHA end-to-end anastomosis	6 (46.1)	Ascites	5 (38.4)
None	1 (7.6)	Pulmonary infection	1 (7.6)
		Vascular anastomotic stenosis	1 (7.6)
**HV reconstruction** (***n*,%)**		**Clavien-Dindo classification** (***n*,%)**	
LHV stump reconstruction	5 (38.4)	I	1 (10)
LHV-MHV venoplasty	7 (53.8)	II	2 (20)
LHV stump plasty - saphenous vein lengthening	1 (7.6)	III	3 (30)
**BD reconstruction** (***n*,%)**		IV	0(0)
Left bile duct reconstruction + common bile duct end-to-end anastomosis	3 (23.07)	V	4 (40)
Left bile duct reconstruction + jejunal Roux-en-Y anastomosis	9 (69.2)	**ICU time** (**days)**	6(3–16)
Bile duct reconstruction + end-to-end anastomosis + jejunostomy	1(7.6)		
None	1(7.6)	Hospital stay(days)	32 (9-77)
**Extrahepatic organ invasion** (***n*,%)**			
Diaphragm	4 (30.8)	**Follow-up time** (**months)**	43(2-63)
adrenal gland	3(23.07)		
Kidney	2(15.4)	death toll (***n*%)**	4 (30.7)
Combined invasion	4 (30.8)		

Note: PHI staging system (HX-PHI Staging system) (9), divided into 3 classes and different types according to the severity of the duct as mentioned earlier violations, of which Class I (P3I1-3H1-2), Class II (P1H2I3, P2H2I3) Class III (P2H1I2, P2H2I2) Class IV (P2H1I1).

**Table 2:** Clinical data and preoperative evaluation of 13 patients with advanced hepatic alveolar echinococcosis.

NO	Gender	Age	BMI	Treatmens	Child-pugh	PNM	RLV	SLV	RLV/SV
							ml	ml	%
1	F	33Y	19.10	PTCD	B	P4N0M0	560	1,118	50.08%
2	F	35Y	20.70	–	A	P4N0M0	520	1,132	45.92%
3	F	42Y	23.88	PTCD	A	P4N0M0	1,300	1,231	105.59%
4	M	32Y	17.72	PTCD	B	P4N1M0	565	1,118	50.53%
5	F	18Y	18.49	PTCD	A	P4N1M0	441	991	44.00%
6	M	47Y	20.49	–	A	P4N1M1	1,015	1,132	89.63%
7	F	39Y	21.49	PTCD	A	P4N1M0	1,338	1,062	126.00%
8	F	47Y	22.21	PTCD	A	P3N1M0	566	1,316	43.00%
9	F	28Y	19.10	–	B	P4N1M0	1,261	1,118	112.80%
10	M	43Y	23.49	PTCD	B	P3N1M0	589	1,182	57.00%
11	F	34Y	19.21	–	A	P4N1M0	784	1,108	51.20%
12	F	42Y	21.24	PTCD	B	P4N1M0	634	1,056	60.04%
13	F	59Y	20.14	–	A	P4N1M0	829	1,243	66.69%

Note: F, Female; M, Male; RLV, Residual liver volume; SLV, Standard liver volume; PTCD, percutaneous transhepatic cholangiography drainage.

### Preoperative surgical feasibility assessment

**Figure 1:**
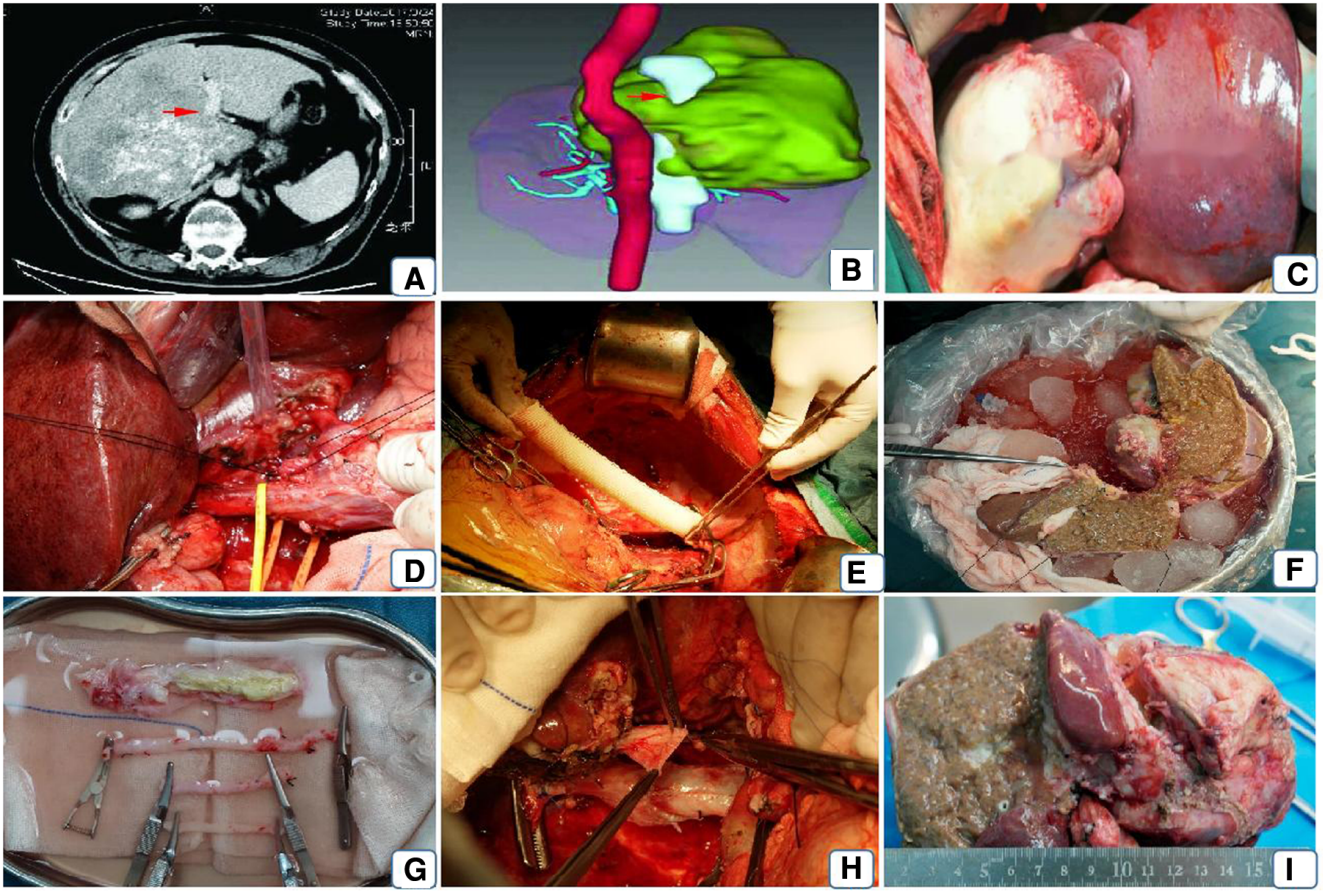
Patient's preoperative imaging assessment and surgical procedure. Note: (**A**) preoperative vesicular encapsulated lesion approximately 17*17 cm in diameter, invading the right branch of the portal vein (red arrow); (**B**) preoperative 3D reconstruction showing the lesion invading the encapsulated inferior vena cava and the left and middle hepatic veins; (**C**) opening the abdominal cavity to expose the normal liver and the lesion tissue; (**D**) dissecting the first hepatic portal, blocking the portal vein, the innominate hepatic artery and the common hepatic duct respectively and sharply dissecting them; (**E**) using artificial vessels connect the suprahepatic and intrahepatic inferior vena cava and end-lateral anastomosis of the portal vein to the artificial vessel; (**F**) ex vivo resection of the lesion and repair of the plastic inflow and outflow tract; (**G**) reconstruction of the inflow, outflow tract or inferior vena cava of the liver using autologous or allogeneic vessels; (**H**) replacement of the artificial inferior vena cava by the isolated free and repaired autologous inferior vena cava; (**I**) resected lesion.

Imaging was performed to understand the size, extent of infiltration,vascular and biliary involvement of the liver lesion (10, 11) (Figure 1A); for patients with extensive invasion and/or compression of the posterior hepatic inferior vena cava, an inferior vena cava angiogram was required; the degree of inferior vena cava stenosis was assessed and the presence or absence of collateral circulation established (Figure 1B); all patients were treated with three-dimensional reconstruction of the liver and a liver model was created (Figure 1C) to visualize the size of the lesion, calculate the actual liver volume, liver lesion volume, remaining liver volume, and standard liver volume to assess the surgical outcome to ensure the safety of the procedure (12, 13); the ICGR 15-minute retention rate was performed 1 week before surgery in all patients to assess the reserve function of the patient's liver to predict the occurrence of liver failure after surgery; all patients were treated preoperatively according to semi-ex Vivo All patients were prepared preoperatively for hepatectomy or hepatectomy combined with autologous transplantation.

### Surgical procedures

After general anaesthesia, an incision is made through a herringbone incision, layer by layer, until the abdominal cavity is entered. Once the lesion is seen, intraoperative ultrasonography is used to demonstrate the intrahepatic invasion of the lesion further, to make a preliminary determination of the status of the lesion to the hilar, and to examine other extrahepatic organs for the presence of extrahepatic metastases. Adequate freeing of the liver, with the the third porta hepatis (at the lower part of the vena cava sulcus, at the outlet of the right posterior inferior hepatic vein and caudate vein) as utterly free as possible or, if the the third porta hepatis is found to be invaded by a lesion, *in vitro* separation after adequate dissection; sequential blocking and dissection of the CHD (common hepatic duct), PHA (Proper hepatic artery), SHIVC (suprahepatic inferior vena cava), IHIVC (Inferhepatic inferior vena cava) RHIVC (Retrohepatic inferior vena cava) from the body (Figure 1D). The inferior vena cava was reconstructed by end-to-end anastomosis of the artificial vessel-superior and inferior vena cava using a 6-0 prolene wire (Figure 1E). A temporary portal vein shunt was established by end-to-end anastomosis of the PV to the artificial IVC. These two routine steps maintain hemodynamic stability during the procedure. Blood gas analysis, including pH, LAC, and SaPO2, was performed at 1-hour intervals, and anaesthetic adjustments were made accordingly. After the liver is removed from the body, the liver is continuously lavaged with 0-4° lavage solution *via* the portal vein, followed by sequential lavage of the hepatic artery and the intra- and extrahepatic bile ducts. The ultrasonic knife and bipolar electrocoagulation forceps were used to separate the liver parenchyma along the hepatic sickle ligament and carefully isolate the liver lesion, followed by complete resection of the diseased liver (Figure 1F), the healthy side intact. After the reserved liver has been repaired ex vivo (Figure 1I), the reconstructed hepatic vein is lateralized to the artificial inferior vena cava or, if the patient's inferior vena cava can be preserved, to the vena cava with an end-to-side anastomosis. If the inferior vena cava is reconstructed outside the body, the artificial IVC is removed from the body at the time of liver transplantation, the autologous reconstructed vessel is connected to the superior and inferior hepatic vena cava, and the transplanted hepatic vein is then anastomosed end-to-end with the IVC (Figure 1G,H). Successive anastomoses are performed on the portal vein and the hepatic artery. If the patient's common bile duct can be preserved, an interrupted pair of common bile duct anastomoses are performed. If it is not possible to preserve, a choledochojejunostomy is performed.

### Postoperative management and follow-up

Postoperative complications were assessed according to Clavien-Dindo classification; all patients were routinely given albendazole for 1 year postoperatively and were followed up by outpatient review and telephone call after discharge, which included the patient's abdominal ultrasound, total abdominal CT, liver function, patient survival and the presence of recurrence, up to December 2020.1.5.

### Statistical analysis

The data were statistically analyzed using SPSS 23.0, with measures expressed as mean, standard deviation (X ± S) or median, and counts expressed as a percentile.

## Results

The age range of the 13 patients who underwent ELRA was 18–59 years, with a median age was 38.3 years. Preoperative PTBD was performed in 6 patients, and TBIL concentrations were reduced to ≤60 umol/ L. ICG retention at 15 min was <10% in 6 patients (46.1%) and ICG R 15 was >10% in 7 patients (53.9%). PHI class I (P3I1-3H1-2) in 4 cases (30.8%), class II (P1H2I3, P2H2I3) in 3 cases (23.07%), and class III (P2H1I2, P2H2I2) in 6 cases (46.1%) (Table 1); There were 4 of HAE lesions invaded the diaphragm (30.8%), 3 of HAE lesions invaded the adrenal gland (23.07%), 2 of HAE lesions invaded the kidney (15.4%) and 4 HAE lesions combined with peripheral multi-organ invasion(30.8%); According to the standard liver volume calculation formula of West China Hospital, we calculated the standard liver volume of all patients (14). The median standard liver volume was 1118 ml (1,085–1,206.5 ml), the median residual liver volume was 634 ml (range 526.5–1,338 ml) and calculated GLV/SLV 43-112%, with a mean of 69.42% (Table 1).

The median weight of the autograft was 845.8 g (619.5–1,020.5 g). To maintain hemodynamic stability during the hepatoless period, 12 patients underwent temporary inferior vena cava reconstruction and portal bypass. 1 patient underwent intra-operative extracorporeal vein-venous diversion, of which 7 patients underwent direct reconstruction of the inferior vena cava using autologous vessels and 4 patients underwent temporary replacement of the inferior vena cava using artificial vessels. In two patients, a PV-subhepatic IV end-lateral anastomosis was used for TPS. In one patient, the inferior vena cava was not reconstructed because of the intraoperative formation of retroperitoneal collateral circulation. No postoperative renal insufficiency or gastrointestinal symptoms were observed. The specific reconstruction modalities of the first and second hepatic hilum after transcatheter repair and plastic surgery are shown in Table 1; the median operation time was 14.5 h (11.5–16.15 h), the median anhepatic phase time was 290 min (257–312.5 min). The median intraoperative blood loss was 1,900 ml (1,300–3,500 ml), the median number of erythrocyte suspensions entered was 7.5 u (6–9u). There were 4 patients had no significant complications during hospitalization, and 9 had complications, including 2 cases of biliary leakage, 4 cases of thoracoabdominal effusion, one case of pulmonary infection, one case of vascular anastomotic stenosis, and 1case of recurrence. 4 patients had complications of Clavien-Dindo classification grade III or higher (Table 1). 4 patients died, with a mortality rate of 30.8%. Ultrasound, CT, and blood indices were examined every 3 months to study liver function and the long-term prognosis of ERAT. All patients were regularly treated with albendazole for at least one year after surgery. The median follow-up was 43 months (2–63 months), and one case developed persistent ascites and hypoproteinemia at 8 months postoperatively, which was considered a small liver syndrome (15). One patient developed a chest wall mass at 36 months postoperatively, considered a recurrence of incisional implantation with a high probability of recurrence (16), and was discharged with surgical excision and improvement.

## Discussion

Hepatic alveolar echinoccosis is a disease that can infect both humans and livestock and is also known as “worm cancer” because of its tumour-like growth pattern (infiltrative growth), for which radical resection is the treatment of choice (17, 18). On the other hand, the lack of medical resources in areas with a high prevalence of worms has deprived these patients of the best opportunity for radical resection in clinical practice. The advent of ELRA has given these patients, unable to undergo radical resection, the opportunity for reoperation (19, 20). At the same time, ELRA, as a challenging liver surgery, poses a severe challenge to the multidisciplinary cooperation and operator skills in each treatment centre due to the complexity of the operation, the high degree of difficulty, the long operation time, and the high trauma.

### Preoperative evaluation

Among the indications for ELRA proposed by scholars at home and abroad in the past (21, 22, 23), one of the more important points is the relationship between lesions and blood vessels. We evaluated the number, size, relationship with surrounding tissues, and liver quality of lesions to ensure safety and effectiveness through preoperative 3D reconstruction techniques (24). This technique is more intuitive and more straightforward than conventional two-dimensional planar CT and allows the liver to be viewed from different angles with the blood vessels (25, 26); thus facilitating the surgical planning of ELRA; in addition, some patients with end-stage HAE have a combination of biliary infiltration leading to obstructive jaundice. Obstructive jaundice or elevated bilirubin impairs the regenerative capacity of the residual liver (26), requiring routine preoperative biliary drainage by PTCD or Endoscopic nasalbileduct drainage (ENBD) to relieve hyperbilirubinemia and biliary obstruction. The procedure's safety is effectively ensured by careful assessment of the surgical operation, liver function, and residual liver volume, combined with delicate surgical techniques.

### Management of anhepatic phase

The management of the anhepatic phase is another critical issue that determines whether the procedure can be performed successfully (25). In previously reported ELRAs (27, 28), veno-venous bypass (VVB) was usually routinely used to control hemodynamic stability during the hepatoless phase; however, in another study (29), complications such as postoperative pulmonary thromboembolism and post-reperfusion syndrome were seen in up to 30% of patients. In our medical centre, 11 patients underwent temporary inferior vena cava reconstruction, one did not, and one patient who underwent extracorporeal vein-venous diversion died postoperatively due to pulmonary embolism; in the patient who did not undergo reconstruction, we found abundant retroperitoneal collateral circulation on preoperative inferior vena cava angiography and no significant hemodynamic fluctuations after intraoperative clamping of the vena cava. Therefore we recommend that all patients undergo routine temporary IVC reconstruction combined with portal shunts for hemodynamic stability and to reduce the incidence of postoperative infections caused by bacterial translocation (30, 31). In addition, hypothermic perfusion is also a core technique for liver protection (29), facilitating the maintenance of vascular patency, flushing of red blood cells and anaerobic metabolites from the transplanted liver, and reducing the extent of mitochondrial and nuclear damage; 12 of the 13 patients at our medical centre were perfused intraoperatively with University of Wisconsin solution (UW fluid) and one with HTK fluid, and the median anhepatic phaseliver-free period time in all cases was 290(257–312.5) minutes, with the longest time being 365 min, with no abnormal blood flow abnormal changes after hepatic blood flow was restored. Therefore, our centre believes that the use of cold perfusion of the liver and temporary inferior vena cava reconstruction combined with portal shunt during the hepatoless period can effectively reduce the hemodynamic instability associated with hepatocellular destruction, gastrointestinal stasis and ischaemic reperfusion injury after liver restoration.

### Reconstruction methods of blood vessels and bile ducts

The reconstruction of ELRA graft vessels and bile ducts is complex; All are individualized protocols (32, 33). Our centre described the characteristics of vascular infiltration in 13 surgical patients concerning the vascular infiltration typing of hepatic vesicular peritoneal PHI proposed by Prof. Wang's team (13) (Table 1). A preliminary reconstruction plan had been made preoperatively according to the degree of vascular infiltration. The reconstruction method was finally determined according to the intraoperative findings. Hepatic artery reconstruction was performed with end-to-end anastomosis of the intrinsic hepatic artery; portal vein reconstruction was performed with lateral graft liver.The biliary tract is reconstructed by Hepatic duct (HD) and common bile duct (CBD) anastomosis,Roux-en-Y hepaticojejunostomy, or a combination of these two approaches,depending on the intraoperative situation. As the inferior vena cava is the most severely invaded, the decision to reconstruct the vena cava is based on the presence or absence of retroperitoneal collateral circulation. Intraoperative ultrasound should be performed at the end of all ductal grafts to detect any stenosis or occlusion (33, 34).

### Management of postoperative complications

Management of postoperative complications: Despite the surgeons' increasing knowledge of ELRA, sophisticated intraoperative operations, and improved preoperative assessment, the incidence of postoperative complications is still high, with biliary leakage being the most common, which can be as high as 46.7% (35, 36, 37), and is thought to be related to longer ischaemic time, infection, and biliary anastomotic stenosis; whereas in our current study cohort, biliary leakage occurred in two cases (15.4%), which is significantly lower than the incidence previously reported. There were 2 cases (15.4%), which was significantly lower than the previously reported incidence, and this was closely related to the thorough preoperative planning and precise intraoperative operations at our medical centre. Four patients (30.7%) with complications of pleural effusion and 342 ascites were considered to be due to ischemia-reperfusion injury 343 to the liver and their symptoms improved after active symptomatic treatment; in one patient, a spherical mass in the chest wall was found 36 months after surgery, and a right subxiphoid-rib arch and a mixed density shadow in the right cardio-diaphragmatic angle were seen on CT plain scan of the chest and abdomen; the author compared the location of the recurrent lesion with the preoperative images and preferred intraoperative incisional implantation metastasis (38).

In summary, for patients with advanced HAE, Ex vivo Liver Resection and Autotransplantation have a broad application prospect. It does not require waiting for a liver source does not require long-term postoperative immunosuppressive drugs. Its economic burden is less than that of allogeneic liver transplantation. However, liver transplantation is a complex, challenging and prolonged procedure with more complications, and the patient's general condition is poor, which can easily lead to various postoperative complications. Therefore, accurate preoperative evaluation of the liver, individualized intraoperative pipeline reconstruction, and delicate surgical operations are vital steps to successful surgery for patients eligible for this surgical modality. At the same time, multidisciplinary consultations should be strengthened when some rare complications arise. Early diagnosis and treatment should be provided to improve the success rate and patient survival after liver transplantation.

## Data Availability

The datasets presented in this study can be found in online repositories. The names of the repository/repositories and accession number(s) can be found in the article/Supplementary Material.
